# AGING DRAG QUEENS: RISKS, REWARDS, EXPRESSION AND PERFORMANCE

**DOI:** 10.1093/geroni/igac059.3031

**Published:** 2022-12-20

**Authors:** Laura Donorfio, Brian Chapman, Kathleen Henneberry

**Affiliations:** University of Connecticut, Waterbury, Connecticut, United States; University of Connecticut, Waterbury, Connecticut, United States; University of Connecticut, Waterbury, Connecticut, United States

## Abstract

This poster presentation will share the exploratory findings of a qualitative research study examining the lived experiences of older drag queens, age 50+. Drag queens are an understudied and underrepresented population in the social science literature (Knutson, Koch, Sneed, Lee, & Chung, 2020; O’Brien, 2018). Older drag queens have yet to be studied in the social science literature. “Drag Queens possess unique characteristics as a subpopulation of the lesbian, gay, bisexual, and transgender (LGBT) community that warrant exploration” (Knutson, Koch, Sneed, & Lee, 2018). A comprehensive literature review identified approximately a dozen research driven articles from 2004 to the present, studying such areas as motivations, obstacles and stressors in becoming and being a drag queen (Hopkins, 2004; Knutson et al., 2021), depression, substance abuse, and resilience (Knutson et al., 2019; Tillewein & Kruse-Diehr, 2021) and the role gender plays via sexuality identities (Taylor & Rupp, 2004, Levitt et al. 2017). This research seeks to better understand how drag expression is integrated with one’s persona and how it interfaces with dragism, coping, and if drag expression can be used as a tool to foster resilience. Using a semi-structured interview protocol and thematic analysis, many themes emerged including the risks and rewards associated with drag expression, the relationship between aging and being a drag performer, and the shift in traditional versus 21st century drag. Lastly, we highlight the implications for gender and LGBTQIA+ theory and plans for future research.

